# Cardiac Sarcoidosis—Diagnostic and Therapeutic Challenges

**DOI:** 10.3390/jcm13061694

**Published:** 2024-03-15

**Authors:** Dennis Korthals, Michael Bietenbeck, Hilke Könemann, Florian Doldi, David Ventura, Michael Schäfers, Michael Mohr, Julian Wolfes, Felix Wegner, Ali Yilmaz, Lars Eckardt

**Affiliations:** 1 Department for Cardiology II, Electrophysiology, University Hospital Münster, 48149 Münster, Germanylars.eckardt@ukmuenster.de (L.E.); 2 Department for Cardiology I, Division of Cardiovascular Imaging, University Hospital Münster, 48149 Münster, Germany; 3 Department of Nuclear Medicine, University Hospital Münster, 48149 Münster, Germany; 4 Department of Hematology, Hemostaseology, Oncology and Pneumology, Division of Pneumology, University Hospital Münster, 48149 Münster, Germany

**Keywords:** cardiac sarcoidosis, inflammatory cardiomyopathy, ventricular arrhythmia, sudden cardiac death, implantable cardioverter–defibrillator, cardiovascular magnetic resonance, positron emission tomography

## Abstract

Sarcoidosis is a multisystem disorder of unknown etiology. The leading hypothesis involves an antigen-triggered dysregulated T-cell-driven immunologic response leading to non-necrotic granulomas. In cardiac sarcoidosis (CS), the inflammatory response can lead to fibrosis, culminating in clinical manifestations such as atrioventricular block and ventricular arrhythmias. Cardiac manifestations frequently present as first and isolated signs or may appear in conjunction with extracardiac manifestations. The incidence of sudden cardiac death (SCD) is high. Diagnosis remains a challenge. For a definite diagnosis, endomyocardial biopsy (EMB) is suggested. In clinical practice, compatible findings in advanced imaging using cardiovascular magnetic resonance (CMR) and/or positron emission tomography (PET) in combination with extracardiac histological proof is considered sufficient. Management revolves around the control of myocardial inflammation by employing immunosuppression. However, data regarding efficacy are merely based on observational evidence. Prevention of SCD is of particular importance and several guidelines provide recommendations regarding device therapy. In patients with manifest CS, outcome data indicate a 5-year survival of around 90% and a 10-year survival in the range of 80%. Data for patients with silent CS are conflicting; some studies suggest an overall benign course of disease while others reported contrasting observations. Future research challenges involve better understanding of the immunologic pathogenesis of the disease for a targeted therapy, improving imaging to aid early diagnosis, assessing the need for screening of asymptomatic patients and randomized trials.

## 1. Introduction

Sarcoidosis remains a medical challenge, as its exact cause and pathogenesis remain elusive despite extensive research. The prevailing theory suggests that environmental antigens cause a dysregulated T-cell-driven immunologic response in genetically predisposed individuals, ultimately giving rise to non-necrotic inflammatory granulomas [1]. These granulomas can manifest throughout the body, causing inflammation and fibrosis in various organs [1]. Cardiac sarcoidosis (CS) usually occurs in conjunction with extracardiac involvement. However, it may also manifest as an isolated manifestation [2,3]. The main cardiovascular manifestations are conduction system involvement with atrioventricular (AV) block, heart failure (HF), and ventricular tachyarrhythmias (VA), which may lead to sudden cardiac death (SCD) (Table 1) [4,5,6,7,8,9].

The diagnosis of CS remains challenging, as evidenced by lack of consensus among available guidelines [10,11,12]. The Heart Rhythm Society (HRS) consensus statement [11] demands histological proof, while the Japanese guideline [12] allow for a diagnosis relying on clinical criteria and advanced cardiac imaging alone. Endomyocardial biopsy (EMB) is considered as the gold standard for e.g., inflammation [11], although both its sensitivity and specificity are limited due to the patchy distribution of cardiac involvement. Electroanatomic mapping (EAM) guided biopsies may help to distinguish CS from other cardiomyopathies such as arrhythmogenic right ventricular cardiomyopathy (ARVC) [13,14].

Advanced imaging techniques such as cardiac magnetic resonance imaging (CMR) and positron emission tomography (PET) have emerged as important diagnostic modalities to differentiate CS from other entities [15,16,17,18]. These imaging modalities are also becoming increasingly important for risk stratification of SCD and guiding immunomodulatory therapy [19,20,21]. Given the high incidence of SCD [22], its prevention is particularly important.

A specific therapy for CS is not available, as the pathogenesis remains poorly understood. Immunosuppression with glucocorticoids is employed to reduce the burden of inflammation, but data regarding its efficacy to prevent progression are limiting and side effects may be severe. This review provides an updated overview of new research developments, emphasizing clinical aspects and advancements in imaging and the management of VA.

## 2. Clinical Manifestations

A significant proportion of patients with CS have an unremarkable medical history before cardiac symptoms first occur (Table 1). Cardiac symptoms are estimated to occur in 5% of patients with systemic sarcoidosis [23]. While extracardiac involvement is typically detected in most cases eventually, isolated CS is reported in 20–25% of patients [3,24,25,26], which is in line with autopsy and MRI studies suggesting an overall underdiagnosed cardiac condition [27,28,29]. Of note, a clinically isolated CS may indicate a worse prognosis compared to CS concurrent with systemic disease [2,30].

**Figure 1:**
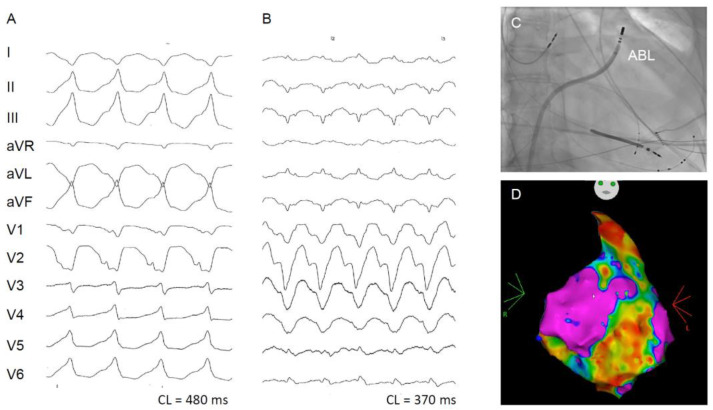
Two monomorphic VT (**A**,**B**) in a 58-year-old female patient with cardiac sarcoidosis and recurrent VT in the presence of significantly reduced RV and LV function, chronic amiodarone therapy and previous implantation of a cardiac resynchronization therapy/defibrillator (CRT-D) system who underwent extensive RV/LV mapping. (**C**) Successful termination of VT (**A**) in the anterior right ventricular outflow tract. ABL (ablation catheter). (**D**) Endocardial voltage map of the RV with extensive low-voltage/scarring (in color 0.5–1.5 mV).

CS is classified as an arrhythmogenic cardiomyopathy, in which inflammation leads to fibrosis. The location and extent of myocardial inflammation determines the course of the disease. Scarring and inflammation promote the formation of reentrant VA. Because inflammation is often multifocal, multiple VA are common [31,32]. Involvement of the right ventricle (Figure 1) seems to indicate an increased risk for VA and may present as a phenocopy of ARVC [13,33,34,35]. As the conduction system is often affected, AV conduction block is a “red flag”, especially in younger patients without known structural heart disease [36]. Supraventricular tachyarrhythmias including atrial fibrillation is rarely observed initially but can present at later stages of the disease [37,38,39]. Signs of heart failure usually indicate left-ventricular infiltration and systolic dysfunction but can also result from diastolic dysfunction and/or restrictive physiology [40,41]. In rare cases, CS can also infiltrate or compress the coronary macro- or microvasculature leading to angina, a diagnosis of myocardial infarction with non-occlusive coronary arteries (MINOCA) or even occlusive myocardial infarction [41,42,43,44].

**Table 1:** Initial clinical manifestations according to a Finish registry study with 289 patients [6].

Clinical Manifestations at Presentation	%
Atrioventricular block, 3rd degree or 2nd degree Mobitz II	46
Sustained ventricular tachycardia	17
Non-sustained ventricular tachycardia or ectopy	7
Aborted sudden cardiac death	4
Heart Failure with reduced LVEF	18
Atrial tachyarrhythmia	1
Syndrome mimicking acute coronary syndrome	4

In up to 50% of cases, AV block and life-threatening VA can present as the first clinical manifestations (Table 1) [45,46]. For a large collective of patients with definite or probable CS, a Finish study revealed a 5-year incidence for SCD of 10%. A significant number of patients (25%) experienced SCD or sustained VA as the first clinical manifestation over a 5-year follow-up [22]. Mainly due to advanced imaging techniques the detection rate of CS has been rising. Kandolin et al. [2] demonstrated a more than 20-fold increase in CS detection rates from 1991 to 2020 in Finland. The prevalence of clinically manifest CS based on data of this large study is estimated to be around 14/100,000 in those over 18 years [2]. Systematic data regarding prevalence of CS in other regions are scarce. In addition, considerable geographical and racial differences between northern and eastern countries exist. Recent observations also suggest that women are disproportionately affected by the disease [9,46].

## 3. Diagnosis

In 2014, the Heart Rhythm Society (HRS) [11] published the first international guideline for the diagnosis and management of CS. Prior to this, the Japanese Ministry of Health and Welfare criteria were published in 1992. These criteria were updated in 2006 and 2016 [12], permitting a diagnosis of CS without histological proof based on a set of clinical criteria and advanced imaging [47].

**Figure 2:**
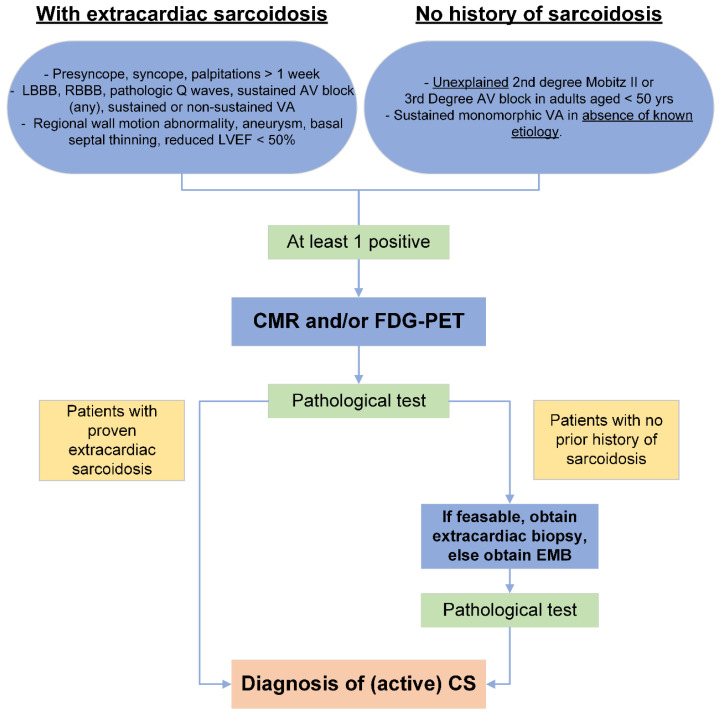
Initial evaluation and selection of patients for advanced imaging, modified from [11]. LBBB: left bundle branch block; RBBB: right bundle branch block; AV: atrioventricular, LVEF: left ventricular ejection fraction; VA: ventricular arrhythmia; CMR: cardiovascular magnetic resonance, FDG-PET: 18-fluordesoxyglucose positron emission tomography; EMB: endomyocardial biopsy.

An early diagnosis of CS remains challenging due to its variable clinical presentation (Table 1) and lack of pathognomonic features. If cardiac symptoms occur in a patient with known extracardiac sarcoidosis further work-up is recommended. High-grade atrioventricular block in otherwise healthy middle-aged individuals should also raise suspicion for CS, with an estimated prevalence of CS between 20 and 30% (Figure 2) [48,49,50]. In a prospective study, the prevalence of CS was also reported to be as high as 28% in patients presenting with monomorphic VT and no prior history of sarcoidosis or ischemic cardiomyopathy [51]. An ECG, Holter-ECG, and cardiac echocardiogram should be acquired at initial presentation. However, initial ECG test findings are often unspecific (Table 2). If extracardiac sarcoidosis has already been confirmed, laboratory analysis of cardiac enzymes such as troponin and natriuretic peptides can support the diagnosis. Angiotensin-converting enzyme (ACE) and soluble interleukin-2 receptor may also be elevated, but the sensitivity of these parameters for the diagnosis of CS is very low [52,53]. The role of echocardiography in the diagnosis of CS is also limited as no pathognomonic echocardiographic features exist. Unspecific findings like regional wall motion abnormalities, wall thickening/thinning, as well as reduced LV longitudinal strain may be present [29,45]. Depending on the results of the initial evaluation (Figure 2), patients with suspected CS should be selected for imaging tests with CMR and 2-deoxy-2-[^18^F]fluoro-D-glucose (FDG)-PET to establish the diagnosis and enable further treatment.

### 3.1. Advanced Imaging: Cardiovascular Magnetic Resonance (CMR)

CMR allows accurate assessment of anatomy and function, but more importantly, it can also depict even subtle structural myocardial changes indicative of fibrosis and/or inflammation [16,21,54]. This is achieved through the utilization of techniques such as late gadolinium enhancement imaging (LGE) [55], T2-weighted (T2w) imaging [56], and T1-/T2-mapping [57]. Cine imaging is employed for evaluating cardiac function. Multiparametric CMR studies have been shown to yield high sensitivity and specificity (>90%) in the diagnosis of CS [29].

**Figure 3:**
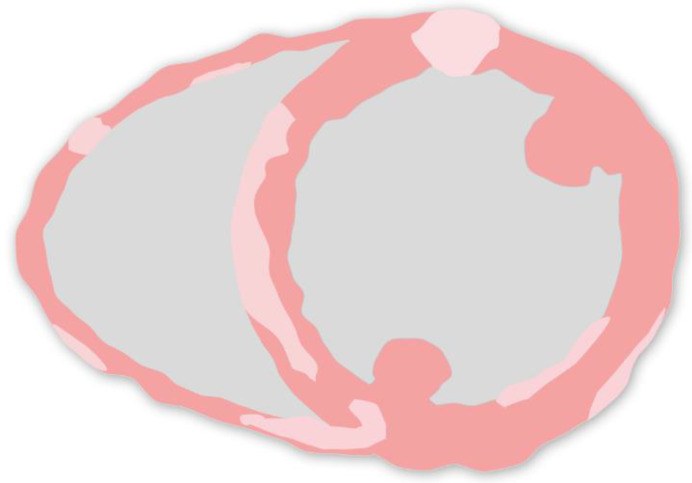
LGE phenotypes in cardiac sarcoidosis; illustration of a short axis view depicting late gadolinium phenotypes of CS. LGE distribution is most commonly multifocal and patchy. It can affect all myocardial layers (subendocardial, midmyocardial, epicardial) and can also present transmurally. LGE is most frequently seen in the basal septum on the RV side as well as anteroseptal but can appear anywhere. A “hook” sign of septal LGE extending to the RV has been described as marker of CS but is also seen in giant cell myocarditis. The presence of RV LGE might be associated with additional risk for VAs compared to LV involvement only.

In patients with suspected CS, LGE-CMR is the key imaging technique for detecting fibrosis and inflammation (Figure 3) [55]. It reveals forms of myocardial damage, which may arise from necrosis and edema during the acute phase as well as fibrosis in the presence of chronic myocardial injury [58]. Overall, the LGE distribution pattern is mostly multifocal and patchy [28,59] but the basal septal wall on the right ventricular (RV) side and the anteroseptal walls are most frequently affected (Figure 3). Involvement may also extend to the inferolateral wall and the inferior RV insertion. Myocardial damage can be limited to the subendocardial layer but may also occur in a transmural or subendocardial pattern. A “hook sign” of septal LGE extending into the RV free wall has been proposed as a sensitive imaging marker [60] but recent studies have also shown this pattern for giant cell myocarditis [61,62].

The presence of LGE is highly prognostic for the occurrence of VA [63]. In a recent systematic review and meta-analysis of 13 studies with a total of 1318 patients with histologically proven sarcoidosis only 1 patient out of 584 with no LGE had VA over a follow-up of 3.1 years, corresponding to a negative predictive value of 99.8%. Conversely, the likelihood of VA occurring in patients with LGE positivity was 20 times higher than for patients without LGE. Furthermore, the presence of biventricular LGE was found to be associated with significantly increased risk of VA [19].

### 3.2. Advanced Imaging: FDG-PET

**Figure 4:**
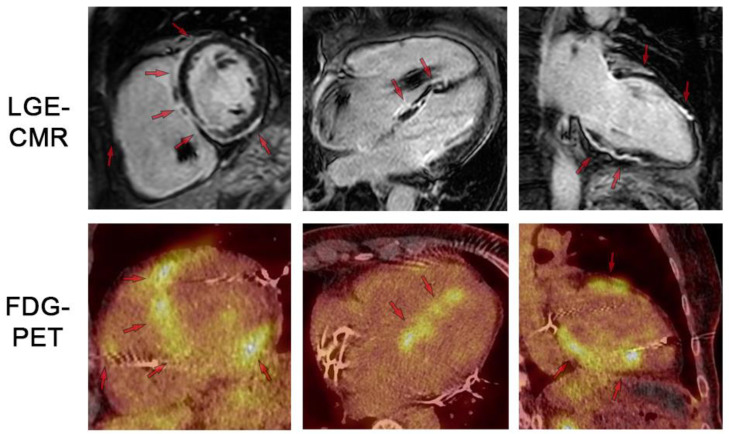
LGE-CMR and FDG-PET images for respective short-axis, 4-chamber, and 2-chamber orientations. The patient was a 65-year-old woman with no prior heart condition initially presenting with third-degree AV block. After a year, the patient developed VT; the pacemaker was subsequently upgraded to an ICD system. The diagnosis of CS was established at this time. Top row shows LGE-CMR fibrosis imaging. Arrows indicate areas of abnormal LGE in a patchy distribution pattern reflecting inflammation and/or scar. Bottom row shows fusion FDG-PET/CT showing patchy uptake in regions of scar, suggesting active inflammation. Note the artifact in the RV cavum caused by the ICD lead. LGE: late gadolinium enhancement. FDG: 18-fluordesoxyglucose positron emission tomography.

FDG-PET serves as a valuable tool for visualizing active inflammation, particularly in the context of CS. FDG is a radiolabeled glucose analog with high uptake in inflammatory cells like macrophages and T-lymphocytes [18,64]. Focal or focal-on-diffuse FDG uptake patterns (Figure 4) indicate active inflammation in CS [18,65]. A characteristic finding is a mismatch pattern, where focal FDG uptake coincides with a perfusion defect. Effective patient preparation is crucial as healthy myocardium naturally exhibits increased physiological FDG uptake. This involves adhering to a high-fat, low-carbohydrate diet that suppresses physiological myocardial glucose consumption. Suboptimal suppression occurs in 10–20% of cases, leading to potentially misleading false positive or inconclusive results [66,67].

Based on insights from a recent meta-analysis [68], sensitivity and specificity are approximately 85%. However, it is important to note that this data are predominantly derived from rather small and mostly retrospective studies, and the reference standard for diagnosing CS relied exclusively on clinical criteria [12], demonstrating significant study limitations. Furthermore, other inflammatory cardiomyopathies (in particular viral or autoimmune forms of myocarditis), systemic rheumatological disease with cardiac involvement und some genetic cardiomyopathies may also cause abnormal or even focal FDG uptake [69]. An inherent advantage of FDG-PET lies in its capability to visualize inflammatory processes not only in the heart but also in other organs. Extracardiac uptake seems to increase the specificity of PET for CS as well as identifying extracardiac manifestations [70]. FDG-PET also plays a key role in guiding immunomodulatory therapy. Many clinicians perform repeat scans to assess response to therapy [71,72,73].

Retrospective studies in patients with known or suspected CS have shown a prognostic role of abnormal cardiac FGT-PET findings [20,74,75]. Nevertheless, it is crucial to note that the quality of available data is relatively low and conflicting results have been reported [76,77]. Most studies have found that an abnormal FDG uptake plus a resting perfusion defect (“mismatch pattern”) is associated with adverse cardiac events, including VA and SCD [20]. Of note is that pathological atrial FDG uptake seems to predict the occurrence of atrial tachyarrhythmia, including atrial fibrillation [38]. Looking ahead, the field is exploring more specific molecular targets for PET, including the fibroblast activation protein (FAP) [78] and somatostatin receptors [79]. These potential targets may provide a more precise assessment and reduce limitations associated with FDG-PET, such as the necessity for physiological suppression of FDG uptake.

### 3.3. Biopsy and Histopathology

Histopathological confirmation of sarcoidosis remains the gold standard. In cases of extracardiac sarcoidosis, opting for a biopsy of lymph nodes or lung tissue is not only safer but also offers higher sensitivity compared to endomyocardial biopsy (EMB) [11,79,80]. We adopt a diagnostic approach where extracardiac histological confirmation of sarcoidosis, coupled with positive CMR and/or FDG-PET findings, is sufficient to diagnose cardiac involvement. If an extracardiac approach is not feasible, we recommend performing EMB after acquiring CMR and/or FDG-PET [81] images, and/or electro-anatomical mapping [13]. An image-(CMR or PET) or EAM-guided biopsy has been shown to improve sensitivity significantly [81,82]. In contrast, sensitivity of non-targeted EMB is at approximately 25% [83,84]. In select cases, and especially if repeat procedures are necessary, we also employ intraprocedural transesophageal echocardiography (TEE) or intracardiac echocardiography (ICE) to improve sensitivity. Given the patchy distribution pattern of sarcoidosis, it is advisable to take several samples to mitigate the risk of sampling errors.

### 3.4. Differential Diagnosis and Screening

Distinguishing CS from other conditions with similar clinical presentations or cardiac imaging findings is crucial. These conditions include myocarditis, various conduction system diseases, genetic cardiomyopathies, and ischemic heart disease. Lyme carditis [85], giant cell myocarditis, and genetic cardiomyopathies such as laminopathies may all present with conduction system disease [21,33,86]. Notably, giant cell myocarditis (GCM) is a rare, but important phenocopy. CMR findings in GCM have been shown to be indistinguishable from CS in a small, blinded register [62]. Many cases can only be differentiated by EMB and even then, the histopathological features of GCM and CS can be similar. This similarity has sparked an ongoing discussion regarding whether CS and GCM are entities within a spectrum of the same underlying inflammatory condition [87,88,89,90]. Of note, the presentation of GCM is usually markedly more acute and fulminant. Like CS, GCM responds to immunosuppression, but the prognosis remains poor [8,91].

ARVC may mimic CS [13,34] and, e.g., epsilon waves may occur in both conditions [13,37]. The presence of AV block favors CS, while epi- or midmyocardial circumferential LGE with LV involvement favors ARVC. Fat infiltration of the RV also favors the diagnosis of ARVC [92]. An electrophysiological study including RV mapping may also be useful to distinguish ARVC from CS in patients with right-sided VA [13,93].

A dilated LV phenotype is common in advanced CS, leading to potential misinterpretation as dilated cardiomyopathy (DCM). Important other differential diagnosis include genetic cardiomyopathies caused by mutations in desmosomal genes (such as desmoplakin) [94] and/or lamin A/C genes [95,96], as they may share clinical characteristics including conduction system disease and VA [21]. In cases where CS is not histologically confirmed and remains unresolved, genetic testing should be considered, especially when only cardiac manifestations are present [86].

Given the high incidence of VA and AV-block as initial cardiac manifestations, with rates possibly as high as 40–50% [45,46], there’s ongoing debate on screening for cardiac involvement in patients with sarcoidosis. ECG and echocardiography lack sensitivity in detecting cardiac involvement. Murtagh et al. [97] screened 201 patients with sarcoidosis but without cardiac symptoms and preserved left ventricular ejection fraction (LVEF) using CMR. 20% of these patients were LGE-positive. During a mean follow up of 36 months, these patients exhibited a twenty-fold risk for VA and SCD, when compared to the LGE-negative cases.

## 4. Management

Management of patients with sarcoidosis is often difficult and multifaceted, especially in patients with multi-organ involvement, calling for multidisciplinary care. The cornerstone of treatment is anti-inflammatory therapy employing non-specific immunosuppression with glucocorticoids. This strategy is fundamental to thwart the progression towards fibrosis. In CS, managing high-grade AV block and VA is crucial, given the high prevalence of SCD. Hence, it is important to evaluate these patients for pacemaker and defibrillator therapy.

### 4.1. Immunosuppression

**Figure 5:**
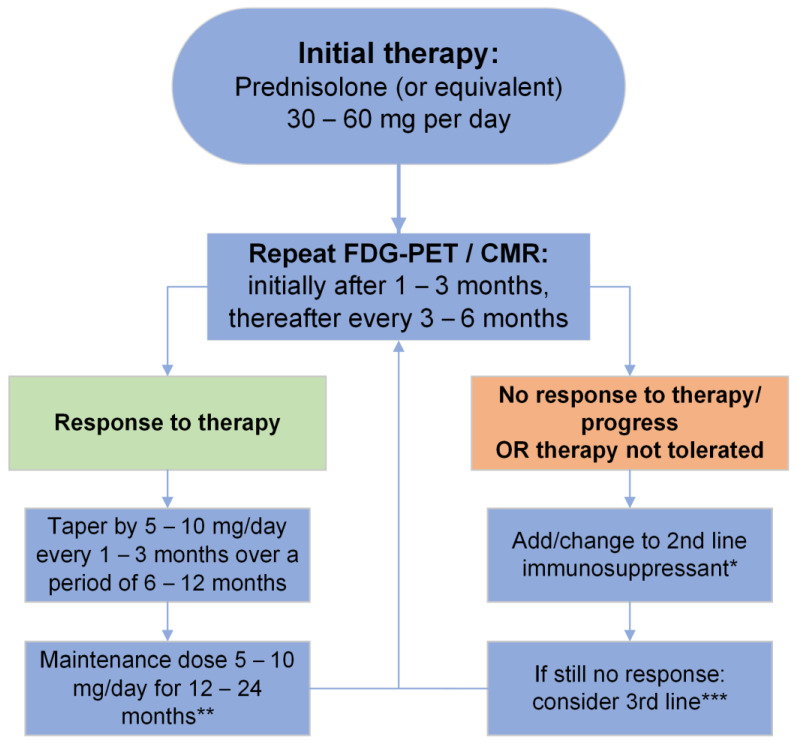
Proposed treatment algorithm for patients with manifest CS, modified from [10]. * Agents: Methotrexate, Azathioprine, Mycophenolate mofetil; ** we terminate therapy guided by advanced imaging findings; *** agents: Leflunomide, TNF-α-antagonists (Infliximab, Adalimumab), Cyclophosphamide, Rituximab.

We recommend immediately initiating glucocorticoid therapy after proof of active myocardial inflammation by advanced imaging (PET or CMR) if a subsequent, immediate, and targeted biopsy is not possible (Figure 2 and Figure 5) [10,11,47]. It is unknown whether asymptomatic patients should be treated as well [98]. In these cases, we opt for shared, individualized decision-making considering the extent of inflammation as well as the side effects of therapy. In general, we favor early therapy even in asymptomatic patients based on indirect observational evidence and a major focus on imaging results [99].

Data regarding the benefits of anti-inflammatory treatment with corticosteroids are limited to small observational studies. In a systematic review of 10 observational studies [100] an improvement of AV conduction was found, but a conclusion on the effectiveness of glucocorticoid therapy on mortality, HF, and VA was not possible. A systematic review of 34 small, mostly retrospective studies involving 1297 patients demonstrated an improvement of AV conduction in about 40% of patients with glucocorticoid therapy. An improvement of LV dysfunction was also observed. However, no conclusions regarding VA or mortality could be drawn [101]. Recently, a prospective, randomized trial of 59 patients refractory to glucocorticoid therapy evaluated the effectiveness of methotrexate (MTX) as add-on therapy over a follow up of 3.3 years [73]. The extent of inflammatory activity was measured in serial FDG-PET scans after initiation of therapy with prednisolone (PSL). Patients were assigned to a poor-response (17%), or response group (83%) based on the measured reduction of inflammatory activity in a FDG-PET follow-up after 6 months. Interestingly, these patients exhibited fewer cardiovascular events (SCD and HF) than the poor-response group. The poor-response patients were randomized to Re-PSL and PSL + MTX groups. Thereafter, the study found no significant difference between these groups regarding reduction of inflammation.

An initial dose of 30 to 60 mg of PSL per day is recommended by most experts [10,11,12]. Predominantly retrospective and monocentric trials indicate there may not be a substantial prognostic advantage for a dosage ≥30 mg compared to <30 mg of PSL per day [102,103]. After initiation of therapy, response to therapy should be re-evaluated by FDG-PET after 3 months [103]. If a patient responds to therapy, the dose should be slowly tapered to 10–15 mg PSL per day during the following 6–12 months. Subsequently, a gradual reduction in dosage every 3 to 6 months may be implemented with a maximum therapy duration of 12–24 months, according to expert opinion (Figure 5) [10]. No data regarding the optimal length of therapy exist. Thus, we base the decision to terminate glucocorticoid therapy on imaging findings. During the first years after discontinuation of therapy, annual follow-up visits including advanced imaging are recommended as late relapses are not uncommon [104]. We extend the intervals for control CMR/PET studies in case of normal findings.

If encountering severe side-effects of PSL therapy, adding MTX as a non-glucocorticoid agent may be discussed in order to decrease the dose of PSL (or even stop PSL treatment) [105,106]. Azathioprine, leflunomide and mycophenolate mofetil, or tumor necrosis factor (TNF) antagonists (adalimumab or infliximab) are second- and third-line non-glucocorticoid options, which need to be employed with caution and require regular follow-up as serious side effects may appear (Figure 5) [107,108,109,110].

Several RCTs are ongoing; the “*Cardiac Sarcoidosis Multi-Center Prospective Cohort (CHASM-CS)*” (https://clinicaltrials.gov/study/NCT01477359 (accessed on 1 February 2024)) [111] trial explores whether a low-dose PSL/MTX regime is non-inferior to a standard PSL dose. The “*Study to Assess the Safety, Tolerability, and Efficacy of Namilumab in Participants with Active Cardiac Sarcoidosis (RESOLVE-Heart)*” (https://clinicaltrials.gov/study/NCT05351554 (accessed on 1 February 2024)) investigates the safety profile of Namilumab targeting the granulocyte-macrophage colony stimulating factor, aiming at modifying active inflammation in CS. “*The Interleukin-1 blockade in cardiac sarcoidosis: study design of the multimodality assessment of granulomas in cardiac sarcoidosis: Anakinra Randomized Trial (MAGiC-ART)*” (https://clinicaltrials.gov/study/NCT04017936 (accessed on 1 February 2024)) [112] is designed to demonstrate a benefit of interleukin-1 blockade on biomarkers and advanced imaging findings. The “*Japanese Antibacterial Drug Management for Cardiac Sarcoidosis (J-ACNES)*” [113] trial investigates the effect of antibiotic treatment in addition to PSL in patients with CS based on the assumption that *Propionibacterium acnes* might play a role in the pathogenesis of CS.

### 4.2. Management of Heart Failure

CS Patients with HF (with preserved or reduced LV function) should receive standard guideline-directed therapy. Data on the effectiveness of PSL therapy on LV dysfunction is inconclusive; one retrospective study reported an improvement of LV function in patients with mild LV dysfunction [99] while another study reported a benefit for patients with severe LV dysfunction but not for patients with mild dysfunction [2]. There are no randomized prospective data available on this topic. However, we propose treatment with glucocorticoids when there is evidence of active myocardial inflammation, according to expert consensus [11].

Mechanical assist devices and heart transplantation should be considered for patients with a fulminant course of disease and/or CS-relative terminal heart failure. Data indicate a good post-transplant survival and a comparable outcome in relation to non-CS transplant recipients [114,115,116].

### 4.3. Management of Arrhythmias and Conduction Disease

High-grade AV block and VA are frequent early manifestations of CS (Table 1) [2]. In patients with manifest CS, an about 10% cumulative risk of SCD after 5 years from presentation, and a 24% composite risk of sustained VA and SCD has been reported [22]. For asymptomatic patients with proven CS the risk is likely lower, but respective data are limited to (small-sized) reports. Therefore, it is important to carefully select patients at risk for timely implantation of a cardioverter-defibrillator (ICD). Guidelines of the European Society of Cardiology (ESC), the HRS, and the American College of Cardiology (ACC) [4,5,11,117] give recommendations regarding treatment of symptomatic VA, risk stratification, and prevention of SCD. In CS patients with a pacemaker indication because of high-grade AV block, the guidelines unanimously recommend evaluation of ICD implantation, regardless of LV function.

### 4.4. Management of Ventricular Arrhythmias

**Figure 6:**
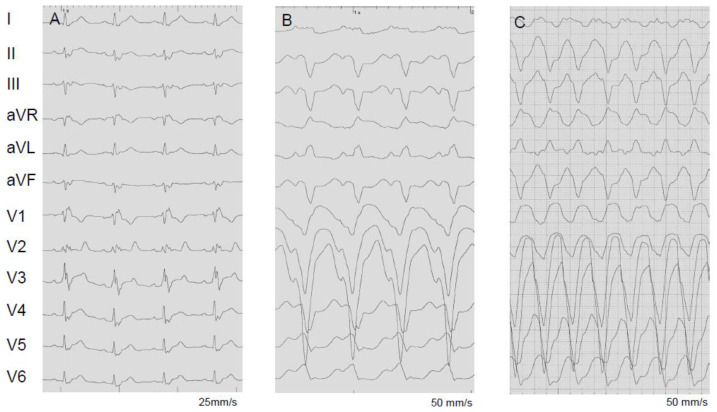
(**A**) Twelve lead ECGs of a 35-year-old male with proven sarcoidosis. Please note the first-degree AV block and the right bundle branch block with a prominent surface area of the maximum R’ wave in leads V1 (see also Hoogendoorn et al. [34]). Monomorphic sustained VT (CL = 400 ms) induced before (**B**) and 6 months after steroid therapy (**C**). Before (**B**) PET demonstrated acute inflammation. This indicates that reduction of inflammation by immunosuppressant therapy may be proarrhythmic. The patient was initially asymptomatic and refused an ICD in (**B**) which was finally implanted after induction of the faster VT (CL = 270 ms) (**C**).

The most common mechanism of VA is scar-related reentry. The management of VA in these patients is particularly challenging as scars are often multifocal and disease course is often dynamic. Intramural and epicardial substrates are common. RV scars are often transmural and may be associated with a higher incidence of VA (Figure 1) [19,31]. Whether active inflammation is proarrhythmic by itself is unknown and data on this topic are conflicting [31]. Consequently, the effect of immunosuppression on the occurrence of VA has not been proven [101,118,119]. Interestingly, a recent prospective, randomized study reported a more frequent occurrence of VA after response to immunosuppression and resolution of inflammation (Figure 6). The authors hypothesized that the anti-inflammatory effect of PSL aggravated the scar formation process during tissue restoration [73].

Antiarrhythmic drugs (AAD), mainly amiodarone and sotalol, can be recommended as for other patients with structural heart disease and VA, but proarrhythmic effects should always be considered [120,121,122,123]. Data on AAD therapy specific to CS are not available. In a prospective study of 42 patients, 43% were refractory to AAD therapy and ultimately required VT ablation [124]. Ablation is recommended for patients refractory to AAD therapy with recurrent sustained VA or ICD shocks [4,5]. Generally, a stepwise approach is recommended, and ablation should always be discussed for electrical storms if VA cannot be controlled by medical therapy alone. Mapping and ablation also present challenges due to extensive scarring and the presence of multiple VA morphologies (Figure 1 and Figure 6) [13]. In many cases, repeat procedures are necessary. In a retrospective study by Kumar et al. [32], VA re-occurred in 63% of cases at 1 year, but control was achievable with fewer AAD. In a systematic review including 83 patients with refractory VA, recurrence was reported at 54% during a mean follow-up of 19.6 ± 13.5 months. Epicardial ablation was required in 18–40% of cases [125]. Of note, in a recent multicentric analysis of 158 patients complete procedural success (no inducible VT post-ablation) was achieved in 54%; 41% of these patients had VA storm pre-ablation that did not reoccur post-ablation in 82% of cases [126]. In conclusion, a significant reduction of arrhythmic burden is achieved in all referenced studies, but the overall recurrence rate is high [13,32,124,125,126,127] As a last resort, patients refractory to ablation and AAD may be considered for bilateral sympathectomy [128].

## 5. Risk Stratification and Prevention of SCD

**Figure 7:**
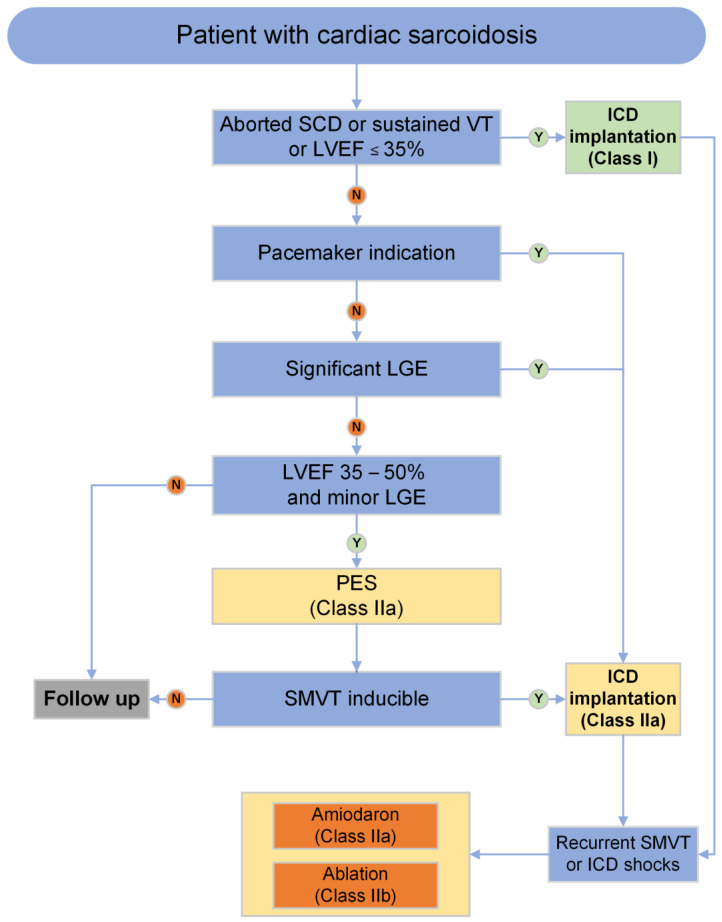
Recommendations for prevention of SCD and management of VA in cardiac sarcoidosis, modified from [4]; SCD: sudden cardiac death, VT: ventricular tachycardia, LVEF: left ventricular ejection fraction, ICD: implantable cardioverter–defibrillator, LGE: late gadolinium enhancement; PES: programmed electrical stimulation; SMVT: sustained monomorphic ventricular tachycardia.

As there is no effective medical therapy for prevention, patients at high risk of VA and SCD should receive ICD therapy (Figure 7). For secondary prevention or when LV function is significantly reduced (LVEF ≤ 35%), all guidelines recommend ICD implantation (Class I). If implantation of a pacemaker is indicated, guidelines recommend ICD implantation regardless of LVEF [4,5,11,117]. A history of syncope is only considered as an ICD indication by the 2017 AHA and 2014 HRS guidelines [4,11,117]. Recent guidelines acknowledge CMR-based LGE as a prognostic marker, recommending implantation regardless of LVEF in the presence of “extensive” or “significant” scar [4,5,117]. Unfortunately, a definition of the latter terms is not provided and there is an ongoing discussion about possible prognostic features of LGE, such as transmurality, location, and extent of LV LGE [19,129]. Debate about the respective choice of possible post-processing methods to define and quantify LGE is also ongoing, especially regarding the question which method reflects the “real” extent of inflammation/scars and has the best prognostic value [130,131]. In a retrospective study by Kazmirczak et al. [132], using the 5 > SD method, a LGE extent of >5.7% in patients with preserved LVEF showed a high annualized event rate for VA/SCD of 12%. Another retrospective study demonstrated a prognostic relevance of qualitative presence of RV LGE regarding VA [133].

The role of electrophysiology study (EPS) is limited to selected patients for risk stratification. Data from observational studies suggest that programmed electrical stimulation (PES) may help to identify patients at risk [134,135]. In a series of 120 patients with proven CS and preserved LVEF undergoing EPS, 6% had inducible VA, of which 43% required ICD therapy during a 4.5 year follow up [136]. Thus, if sustained VA are inducible with PES and LV dysfunction is present, the ESC guidelines also recommend ICD implementation (Figure 7) [4,5].

## 6. Prognosis

Outcome of CS is largely determined by the extent of myocardial infiltration, which can be assessed by CMR-based LGE and FDG-PET (at least in the acute phase). Indeed, LGE has been demonstrated to be a reliable prognostic factor in various studies [19,137]. Reduced LV or RV function and an impaired longitudinal strain have also been demonstrated to be relevant prognostic markers and indirectly reflect the extent of infiltration. Especially the extent of LV dysfunction has been shown to be a strong predictor of risk [98,137]. Clinically, an initial presentation with sustained VA points to a poor outcome [9,137]. Also, an isolated cardiac presentation has been associated with increased risk and adverse outcome [2,3,30]. This likely reflects the presence of advanced disease and relevant myocardial fibrosis in this population, increasing the risk for adverse events such as VA and HF.

The survival rate in patients with manifest CS is around 90% after 5 years and between 80 and 90% after 10 years [9,22,137]. Patients with low LVEF, high levels of brain natriuretic peptide (BNP), prior history of VA with the need for ablation therapy were identified as a high-risk population in a recent, large Japanese registry study [137].

For asymptomatic patents with CS, a study by Murtagh et al. [97] reported that out of 205 patients with preserved LVEF and no ECG abnormalities, 6% had a cardiac event during a 36-month follow up. Interestingly, in a study by Rosenbaum et al. [7], patients with high-grade AV block or VA and advanced imaging findings suggestive of CS but without histological proof were defined as “probable” CS. This collective was compared to patients with histologically proven CS, meeting HRS criteria for diagnosis. No difference in the primary endpoint of hospitalization-free and overall survival at 10 years was found in this study. This study points out an important limitation of current guidelines, which demand histological proof for diagnosis.

## 7. Future Developments

As the etiology of CS remains poorly understood, gaining more insight into immunological pathogenesis remains one of the major challenges. Today, experimental models [1] are at a very early stage due to the very complex nature of the disease and do not mimic all aspects of sarcoidosis sufficiently. Progress would have a major impact on diagnosis and therapy and is therefore an overarching goal of future research efforts. Advancements in cardiac imaging might enable an earlier and more accurate diagnosis. New radiotracers (such as FAPI) may allow highly specific imaging of fibrosis activity [78,138]. Lastly, large multi-center clinical trials are necessary to improve risk stratification, to clarify whether screening of asymptomatic patients with extracardiac sarcoidosis is beneficial, and to investigate the effect of immunosuppression in CS.

## Figures and Tables

**Table 2:** ECG parameters (except AV block) in patients with CS, modified from Willy et al. [37].

ECG Parameters in Diagnosis of CS
Fragmented or prolonged QRS
Bundle branch block
QTc dispersion
T-wave abnormalities (alternans, inversion)
Increased Tpeak–Tend interval
Signal-averaged ECG
